# 

**Published:** 1918-09-07

**Authors:** 


RATES OF SUBSCRIPTION:

Twelve months, 10/10; Six months, 5/5; Three months, 2/9, post free to any address in the United Kingdom. Abroad, Twelve months, 13/-; Six months, 6/6; Three months, 3/3, post free. **THE HOSPITAL "Index" to Volume LXI., October 1917 to March 1918, is now ready, price 3½d. per copy, post free.** Address The Manager, THE HOSPITAL, "The Hospital" Building, 28/29 Southampton Street, Strand, London, W.C. 2.

**Publications**—*continued.*

### J. & A. Churchill. STUDENTS' BOOKS

With 513 Magnificent Illustrations and 20 Coloured Plates. **27s.** net; postage 9d.

### EDEN & LOCKYER'S GYNÆCOLOGY.

*"A book which is the best of its particular kind known to us—certainly in this country, and probably in the English language . . . a book which ought to spring at once into first place amongst British text-books of Gynæcology; a book which may be confidently recommended to both students and practitioners, and one that no Gynæcologist, in particular no teacher of the subject, can afford to neglect."*—BRITISH MEDICAL JOURNAL.

FOURTH EDITION. 354 Illustrations. **18s.** net; postage 9d.

### EDEN'S MANUAL OF MIDWIFERY.

*"We have nothing but praise for this book; a most excellent text-book."*—THE LANCET.

With 74 Illustrations, some in Colours. **15s.** net.

### MEDICAL DIAGNOSIS.

By **ARTHUR LATHAM,** M.D.Oxon., F.R.C.P.Lond., Physician and Lecturer on Medicine, St. George's Hospital; and **J. A. TORRENS,** M.B., B.S.Lond., M.R.C.P.Lond., Assistant Physician, St. George's Hospital.

*"A valuable handbook conveniently arranged; efficiently illustrated."*—EDINBURGH MEDICAL JOURNAL.

*NEW EDITION.*

ELEVENTH EDITION. With 12 Plates, 85 Text-figures. **24s.** net; postage 9d.

### TAYLOR'S MEDICINE.

By **Sir FREDERICK TAYLOR, Bart.,** M.D., President of the Royal College of Physicians.

*"Sir F. Taylor, as a teacher of long experience, sets forth the present state of knowledge clearly and briefly . . . The volume is well balanced, exactly adapted to its purpose, and fully up to date."*—BRITISH MEDICAL JOURNAL.

### HALE WHITE'S MATERIA MEDICA. *NEW EDITION.*

**WITH OFFICIAL WAR REGULATIONS.**

SIXTEENTH EDITION. **8s. 6d.** net.

*"It is fortunate that the continued sale of this essential handbook, even in war time, has made it possible to bring out a new edition. . . . The book is a safe guide to war time as to peace time practice."*—THE LANCET.

### JELLETT'S

**Midwifery** — 7th Edition. 4 Col. Plates. 207 Text-figures. **12s. 6d.** net.

**Gynæcology** — 4th Edition. 11 Col. Plates. 374 Text-figures. **21s.** net.

**Gynæcology, Short Practice of.** — 4th Edition. 10 Col. Plates. 316 Text-figures. **15s.** net.

FIFTH EDITION. 1182 Illustrations, many in Colour. **30s.** net.

### MORRIS'S HUMAN ANATOMY.

Edited by **C. M. JACKSON,** M.S., M.D., Professor of Anatomy, University of Minnesota.

*"A brilliant success, and authors, editors, and publishers deserve to be congratulated heartily on producing an exhaustive treatise on anatomy which is quite one of the best that has been written in the English language."*—THE LANCET.

45 Text-figures. 13 Plates. **12s. 6d.** net.

### PANTON'S CLINICAL PATHOLOGY.

By **P. N. PANTON,** M.A., M.B.Cantab., Clinical Pathologist to the London Hospital.

*"This work strikes us as one which is likely to fulfil its purpose thoroughly, and the printers and publishers, as well as the author, are to be congratulated. The plates, most of which are coloured, are beautifully reproduced. They illustrate the blood in health and in various diseases, blood parasites, absorption spectra, bacteria, cells of puncture fluids and urinary deposits."*—GLASGOW MEDICAL JOURNAL.

SECOND EDITION. 566 Illustrations, 10 in Colour. **21s.** net.

### STARLING'S PRINCIPLES OF PHYSIOLOGY.

By **ERNEST H. STARLING,** M.D., F.R.C.P., F.R.S., Jodrell Professor of Physiology in University College, London.

*"The book has a special value for the medical practitioner, as well as for the student who has to face examinations in physiology. . . . There is nothing of the cut-and-dried formalism so common in scientific text-books about this volume, which is characterised by a broad outlook on the problems of physiology and an unusual readiness to fall back on other kindred sciences in the endeavour to bring these problems towards a satisfactory solution. The book should be widely read by those for whom it is written, and we wish it all success."*—BRITISH MEDICAL JOURNAL.

SECOND EDITION. **6s. 6d.** net.

### MEDICAL and PHARMACEUTICAL LATIN.

By **REGINALD R. BENNETT,** B.Sc.Lond., F.I.C., Late Lecturer in Pharmacology, University College Hospital, London.

FOURTH EDITION. 183 Illustrations. **7s. 6d.** net.

### CORBIN & STEWART'S PHYSICS AND CHEMISTRY FOR MEDICAL STUDENTS.

With 219 Original Illustrations, many in Colour. **21s.** net.

### ANATOMY OF THE HUMAN SKELETON.

By **J. E. FRAZER,** F.R.C.S.

*"We consider the underlying principle of the Book is sound and commendable, and that the author has succeeded admirably in his aim. . . . We would express our gratitude for both the originality and boldness of many of his diagrams."*—BRITISH MEDICAL JOURNAL.

**London: J. & A. CHURCHILL, 7 Great Marlborough Street, W. 1.**

THE first number of the Seale Hayne Neurological Studies, published by Mr. Humphrey Milford, Oxford University Press, will be followed by issues every two months. The editor is Major Arthur F. Hurst, M.D., F.R.C.P., officer-in-charge of the Seale Hayne Military Hospital, Newton Abbot, assisted by Captain J. L. M. Symns, Captain W. R. Reynell, and Lieutenant S. H. Wilkinson. The first part contains six studies on hysteria, and articles on the rapid cure of hysterical symptoms in soldiers and on war contractures.

